# Are neurological complications of monoclonal gammopathy of undetermined significance underestimated?

**DOI:** 10.18632/oncotarget.13861

**Published:** 2016-12-10

**Authors:** Normann Steiner, Angelika Schwärzler, Georg Göbel, Wolfgang Löscher, Julia Wanschitz, Eberhard Gunsilius

**Affiliations:** ^1^ Department of Internal Medicine V (Hematology and Medical Oncology), Medical University of Innsbruck, A-6020 Innsbruck, Austria; ^2^ Department of Neurology, Medical University of Innsbruck, A-6020 Innsbruck, Austria; ^3^ Department of Medical Statistics, Informatics and Health Economics, Medical University of Innsbruck, A-6020 Innsbruck, Austria

**Keywords:** monoclonal gammopathy of undetermined significance, MGUS, MGUS associated neuropathy, multiple myeloma

## Abstract

**Objectives:**

Monoclonal gammopathy of undetermined significance (MGUS) is a premalignancy preceding multiple myeloma (MM) or related disorders. Neurological symptoms caused by the monoclonal immunoglobulins or free light-chains are often associated with a high morbidity. We analyzed the prevalence of neuropathy, clinical features and the long-term outcome in 223 patients (pts.) with MGUS.

**Patients and Methods:**

Between 1/2005 and 3/2015, 223 adult pts. with MGUS were identified in our database.

**Results:**

In36/223 pts. (16%) a neuropathy was diagnosed (MGUS associated neuropathy, MGUS-N). 20 pts. (55%) had a distal symmetric axonal neuropathy, 10 pts. (28%) had a chronic inflammatory demyelinating polyneuropathy and 6 pts (17%) a distal acquired demyelinating symmetric polyneuropathy. In MGUS-NN (without neuropathy) and in MGUS-N, progression to smoldering MM, MM or Waldenstrom's macroglobulinemia (WM) occurred in 17% of the pts. The Immunoglobulin subtype was predominantly IgG in MGUS-NN and IgM in MGUS-N and ≥5.5% plasma cells in the bone-marrow predicted progression to MM and AL-amyloidosis in MGUS-NN and to WM in MGUS-N (p<0.05).

**Conclusion:**

Due to the substantial prevalence of neuropathies, MGUS pts. should be monitored carefully and referred to a specialized center if neurological symptoms occur.

## INTRODUCTION

Monoclonal gammopathy of undetermined significance (MGUS) is a premalignant disorder with a 0.5-1.5% per year risk of progression to multiple myeloma (MM) or other related hematological malignancies [1, 2]. According to the International Myeloma Working Group (IMWG), MGUS is characterized by a monoclonal (M)-protein in the serum of <30 g/l, a clonal plasma cell count in the bone marrow of <10%, and the absence of clinical symptoms [3]. Risk factors for a progression include an M-protein >15 g/l, an abnormal ratio of free kappa (κ) and lambda (λ) light chains, and the non-IgG isotype [4].

MGUS associated neuropathies (MGUS-N) are heterogeneous with respect to the clinical presentation and the underlying pathophysiology and can be caused by deposition of immunoglobulins or amyloid as well as through the interaction with specific antigens on peripheral nerves. Although the prevalence of neuropathy among MGUS patients (pts.) varies considerably in the literature and the identification often depends on patient selection and diagnostic procedures, it is estimated at about 17% [5–7]. Vice versa, 5-10% of pts. investigated for neuropathy have a monoclonal gammopathy [8].

There are three major forms of neuropathy in paraproteinemic disorders: axonal sensory-motor neuropathy, chronic inflammatory demyelinating polyneuropathy (CIDP), and distal acquired demyelinating symmetric (DADS) polyneuropathy. Axonal neuropathy usually presents with sensory symptoms (paresthesia, dysesthesia, anesthesia, neuropathic pain) of distal lower limbs and slowly evolving motor weakness in a length-dependent fashion. It may be associated with IgG/A/M MGUS, but the causal link between the serum paraprotein and axonal nerve damage remains elusive in many cases, although severe pain and autonomic dysfunction may raise the suspicion of amyloidosis [6]. In the demyelinating entities CIDP and DADS a causal relationship with monoclonal gammopathy is considered likely [6, 9]. CIDP is a relapsing or progressive immune mediated neuropathy with proximal and distal weakness and sensory deficits of upper and lower limbs and 22-30% of CIDP pts. are described to have MGUS, commonly IgG or IgA subtypes [10–12]. DADS neuropathy is characterized by predominant distal sensory impairment, ataxia and often tremor, but little or no weakness and has a close association with IgM kappa monoclonal gammopathy that is present in about two-thirds of pts. [13]. In 50-67% of these pts. the IgM monoclonal protein binds to myelin-associated-protein (MAG) [13, 14] causing a characteristic widening of myelin lamellae in nerve biopsies [15]. Despite potent agents in the treatment of pts. with MGUS associated neuropathies, e.g. immunomodulatory agents, plasmapheresis or monoclonal antibodies, some pts. may still present with a high morbidity [9].

The aim of this retrospective single center analysis was to describe the prevalence of neurological manifestations in MGUS pts. and to compare clinical features and risk factors for disease progression in MGUS pts. with and without neuropathy.

## RESULTS

### Patient characteristics

223 pts. fulfilled the criteria for MGUS according to the International Myeloma Working Group (IMWG) criteria, thereof 187 pts. had a MGUS without (MGUS-NN; 84%) and 36 showed a MGUS associated with neuropathy (MGUS-N; 16%). Table 1 summarizes demographic data and laboratory features of MGUS-NN and MGUS-N pts.

**Table 1:** Demographic data and laboratory features at diagnosis; comparison of the two cohorts MGUS-NN and MGUS-N

Parameter	MGUS-NN		MGUS-N	
	N=187	%	N=36	%
Median age (range), years	68 (26-97)		64,5 (42-82)	
Sex f/m				
F	**92**	49*	**7**	19*
M	**95**	51*	**29**	81*
Ig heavy chain (serum)				
IgG	**137**	74*	**17**	47*
IgM	**27**	14*	**13**	36*
IgA	17	9	6	17
biclonal gammopathy				
IgG+IgA	4	2	0	0
IgM+IgG	2	1	0	0
Ig light chain (serum)				
Kappa	106	57	21	53
Lambda	62	33	16	39
Both	10	5	2	5
Not measurable	9	5	1	3
Total protein >UNV (8 g/dl)	12	6	3	8
β-2 microglobulin >UNV	68	43	12	39
LDH >UNV	33	18	4	11
Creatinine ≥1.3 mg/dl	41	22	5	14
Serum calcium >UNV	6	3	0	0
Haemoglobin ≤12 g/dl	**64**	34*	**5**	14*
Platelets <100,000/mm^3^	18	10	4	11
Osteolytic bone lesion	6	3	1	3

MGUS-NN, monoclonal gammopathy of undetermined significance without neuropathy; MGUS-N, monoclonal gammopathy of undetermined significance associated neuropathy; N, number of patients; Ig, Immunoglobulin; UNV, upper normal value; LDH, lactate dehydrogenase.

* p-value <0.05.

Median age at diagnosis was 68 years (range 26-97 years) in the MGUS-NN group and 64 years (range 42-82 years) in the MGUS-N group, respectively. Sex ratio was similar in MGUS-NN pts. (female n=92, 49%; male n=95, 51%), while in the MGUS-N group significantly more pts. were male (female n=7, 19%; male n=29, 81%; p<0.05). In the MGUS-NN cohort more IgG isotype was present than in MGUS-N pts. (n=137, 74% vs. n=17, 47%; p<0.05), whereas more MGUS-N pts. had an IgM isotype (n=13, 36% vs. n=27, 14% in MGUS-NN; p<0.05). No significant differences in the prevalence of kappa (κ) or lambda (λ) light chains were observed. Concerning other laboratory findings, significantly more MGUS-NN pts. had anemia (n=64, 34% vs. n=5 14% in MGUS-N; p<0.05). More MGUS-NN pts. had renal dysfunction (n=41, 22% vs. n=5, 14% in MGUS-N; p>0.05), elevated calcium levels (n=6, 3% vs. 0% in MGUS-N; p>0.05), elevated β2-microglobulin levels (n=68, 43% vs. n=12, 39% in MGUS-N; p>0.05), higher levels of lactat dehydrogenase (LDH) (n=33, 18% vs. n=4, 11% in MGUS-N; p>0.05), and a higher incidence of pre- or coexisting second tumors (n=57, 31% vs. n=8, 22% in MGUS-N; p>0.05). In the total study population 17 pts. (11%) with IgG-MGUS, 6 pts. (22%) with IgA-MGUS, 13 pts. (31%) with IgM-MGUS had neuropathy, but none had biclonal MGUS.

In the MGUS-N group 20 pts. (55%) had an axonal neuropathy, 10 pts. (28%) a CIDP, and 6 pts (17%) presented with a DADS polyneuropathy. Anti-myelin-associated glycoprotein (MAG) antibodies were identified in 4 pts. with IgM-MGUS and demyelinating neuropathy (3 DADS and 1 CIDP phenotype). Table 2 specifies clinical symptoms and severity of neuropathy, associations with immunoglobulin isotypes and treatment modalities in MGUS-N pts. Severity of neuropathy was graded by review of pt. records as mild (sensory symptoms and/or mild distal weakness without impairment of walking), moderate (weakness and/or ataxia interfering with walking) or severe (walking with aid or wheelchair-bound).

**Table 2:** Clinical characteristics of patients with MGUS associated neuropathy

MGUS-N N=36 (100%)			
	axonal neuropathy	CIDP	DADS
N (%)	20 (55)	10 (28)	6 (17)
Sex f/m			
f (%)	5 (14)	2 (6)	0 (0)
m (%)	15 (42)	8 (22)	6 (17)
Ig isotype			
IgG	10 (28)	7 (19)	0 (0)
IgM	6 (17)	2 (6)	5 (14)
IgA	4 (11)	1 (3)	1 (3)
Treatment			
Steroids	3 (8)	9 (25)	2 (6)
IVIG	4 (11)	10 (28)	4 (11)
Rituximab	2 (6)	3 (8)	3 (8)
Azathioprine	0 (0)	2 (6)	0 (0)
Mycophenolate-Mofetil	0 (0)	2 (6)	0 (0)
Lenalidomide	0 (0)	1 (3)	1 (3)
Carfilzomib	0 (0)	0 (0)	1 (3)
Plasmapheresis	0 (0)	2 (6)	0 (0)
Pain therapy	8 (22)	7 (19)	3 (8)
Pain			
Yes	10 (28)	9 (25)	4 (11)
No	10 (28)	1 (3)	2 (6)
Symptoms of neuropathy			
Motor, only	3 (8)	0 (0)	0 (0)
Sensory, only	8 (22)	0 (0)	1 (3)
Sensorimotor	8 (22)	10 (28)	5 (14)
Tremor	6 (17)	3 (8)	2 (6)
Not classified	1 (3)	0 (0)	0 (0)
Severity of neuropathy			
Mild	2 (6)	1 (3)	1 (3)
Moderate	1 (3)	4 (11)	2 (6)
Severe	1 (3)	5 (14)	3 (8)
Not classified	16 (44)	0 (0)	0 (0)

DADS, distal acquired demyelinating symmetric neuropathy; CIDP, chronic inflammatory demyelinating polyneuropathy; Ig, Immunoglobulin; IVIG, Intravenous immunoglobulin.

### Clinical outcome in MGUS-NN and MGUS-N pts

**Figure 1:**
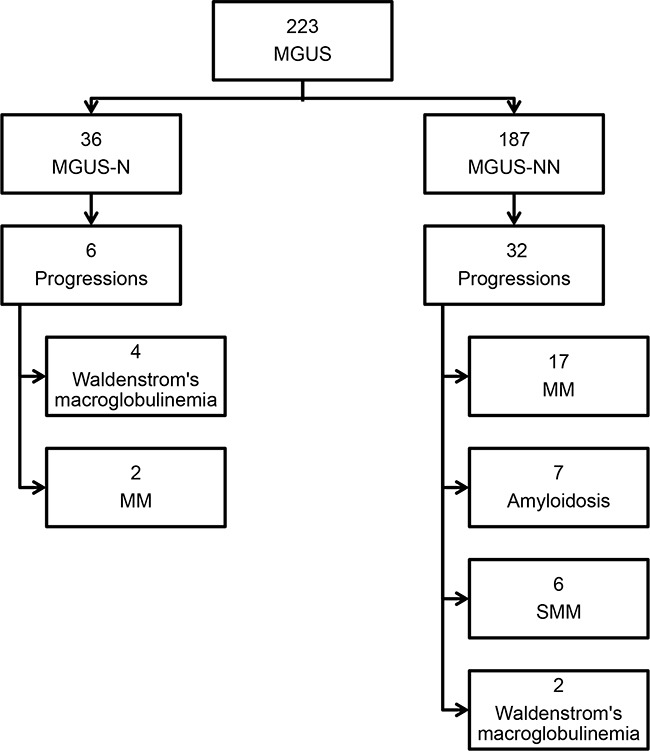
Study population overview MGUS-NN, monoclonal gammopathy of undetermined significance without neuropathy; MGUS-N, monoclonal gammopathy of undetermined significance associated neuropathy; SMM, smoldering multiple myeloma; MM, multiple myeloma.

Figure 1 shows an overview of the study population with regard to the progression to MM or other lymphoproliferative disorders. A total of 38 pts. (17%) progressed after a median time to progression of 48 months (range 2-276 months). 6 pts. progressed in the MGUS-N cohort, thereof 4 pts. to Waldenstrom's macroglobulinemia (67%) and 2 pts. to MM (33%). In the MGUS-NN cohort, a total of 32 pts. progressed, thereof more than half of the pts. to MM (n=17; 53%), 6 pts. to smoldering MM (19%), 7 pts. to AL-amyloidosis (22%), and 2 pts. progressed to Waldenstrom's macroglobulinemia (6%). Comparing the two cohorts MGUS-NN and MGUS-N, the rate of progressions was identical in the two groups (17%). However, significantly more progressions to Waldenstrom's macroglobulinemia were observed in MGUS-N pts. (67% vs. 6%; p<0.05), and significantly more progressions to MM and AL-amyloidosis were seen in the MGUS-NN cohort (53% vs. 33% and 22% vs. 0%, respectively; both p=<0.05). Information on pts. who had a disease progression is shown in Table 3.

**Table 3:** Characteristics of persons who progressed to SMM, MM, AL-amyloidosis or Waldenstrom's macroglobulinemia

ID	Sex	Age	M-protein and free light chains(serum)	Neuropathy	Progression	TTP	2nd Tumor	LFU status
1	m	73	IgAλ	-	SMM	48	-	dead
2	f	43	IgGλ	-	MM	276		alive
3	f	66	IgGκ	-	SMM	58	-	alive
4	m	63	IgGκ	-	AL-amyloidosis	114	-	
5	m	66	IgMκ	Axonal neuropathy	Waldenstrom's macroglobulinemia	13	-	alive
6	f	72	IgGκ	-	MM	159	Skin-CA (squamous cell CA)	alive
7	f	62	IgGκ	-	MM	27	-	alive
8	f	78	IgMκ	-	Waldenstrom's macroglobulinemia	106	-	alive
9	m	58	IgGλ	-	MM	63	Meningeoma	alive
10	m	66	IgGκ	-	MM	2	-	dead
11	m	74	IgAκ	Axonal neuropathy	MM	99	-	dead
12	f	56	IgGλ	-	AL-amyloidosis	10	-	dead
13	m	61	IgMλ	DADS	Waldenstrom's macroglobulinemia	10	-	alive
14	f	77	IgGλ	-	MM	35	-	alive
15	m	65	IgAκ	-	MM	112	-	alive
16	m	60	IgGκ	-	AL-amyloidosis	230	-	dead
17	m	78	IgGκ	-	MM	8	Prostate-CA	alive
18	f	81	IgGλ	-	MM	19	Meningeoma	alive
19	f	76	IgGλ	-	MM	68	-	alive
20	m	65	IgAλ	-	AL-amyloidosis	4	-	dead
21	f	49	IgGκ	-	MM	58	-	dead
22	m	75	IgMκ	Axonal neuropathy	Waldenstrom's macroglobulinemia	91	Urothelial-CA+ Lung-CA	dead
23	m	70	IgGκ	-	SMM	68	-	alive
24	f	62	IgAκ	-	MM	34	-	alive
25	m	67	IgGλ	-	AL-amyloidosis	73	Prostate-CA	alive
26	f	65	IgAκ	-	SMM	158	-	alive
27	m	65	IgGλ	-	AL-amyloidosis	35	Prostate-CA	dead
28	f	85	IgGκ	-	AL-amyloidosis	35	Urothelial-CA	dead
29	m	61	IgGκ	-	MM	41	Prostate-CA	alive
30	m	66	IgGκ	-	SMM	9	Sezary-Syndrome	dead
31	m	84	IgGκ	-	SMM	7	-	dead
32	m	66	IgGλ	-	MM	11	-	alive
33	f	54	IgGλ	-	MM	54	-	alive
34	f	66	IgGκ	-	MM	248	Meningeoma	alive
35	f	58	IgMκ	-	Waldenstrom's macroglobulinemia	163	-	alive
36	m	61	IgGκ	CIDP	MM	12	-	alive
37	f	48	IgGκ+λ	-	MM	272	Gastric-CA	alive
38	m	57	IgMκ	DADS	Waldenstrom's macroglobulinemia	3	-	alive

TTP, time to progression in months; LFU, last follow-up; DADS, distal acquired demyelinating symmetric neuropathy; CIDP, chronic inflammatory demyelinating polyneuropathy; SMM, smoldering multiple myeloma; MM, multiple myeloma; CA, Carcinoma.

### Survival and time to progression (TTP) in MGUS-NN and MGUS-N pts

**Figure 2:**
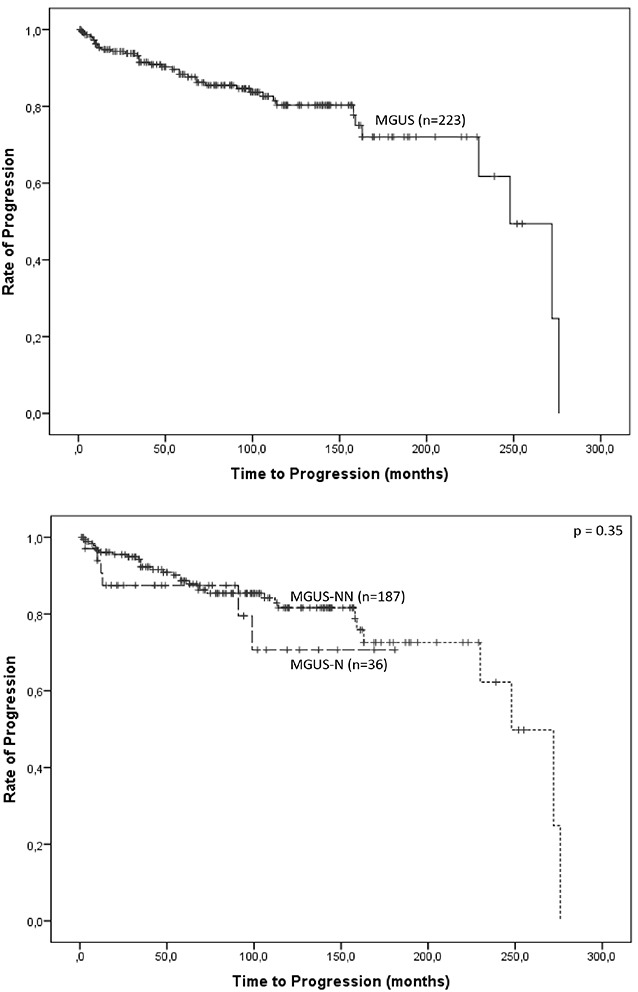
Kaplan-Meier plots on progression for the whole cohort (n=223), and stratified for MGUS-NN and MGUS-N

Median follow-up for the whole cohort (n=223) was 79 months and evaluated patient-years were 1,595. Median overall survival (OS) for the total study population was 255 months. In the progressed 38 pts. a progression rate in the first year of 26% was seen. The progression rate was 1% per year in the MGUS-N cohort and 0.5% per year in the MGUS-NN cohort. Median TTP in the cohort that progressed (MGUS-NN and MGUS-N; n=38) was 48 months (95% CI 20-76). TTP was longer in the MGUS-NN group than in the MGUS-N cohort (median TTP 54 months, 95%CI=30-78 vs. median TTP 12 months, 95%CI=8-16; p>0.05). Kaplan-Meier plots on progression for the whole cohort (n=223) and stratified for MGUS-NN and MGUS-N are shown in Figure 2.

### Risk factors for disease progression

Clinical data of the two cohorts, MGUS-NN and MGUS-N, were evaluated in terms of risk factors for disease progression.

Progressed pts. with MGUS-NN (n=32) showed a significantly higher incidence of IgG isotype (n=25, 78% vs. n=1, 17% in MGUS-N; p<0.05), whereas MGUS-N pts. with progressive disease (n=6) had significantly more IgM isotype (n=4, 67% vs. n=2, 6% in MGUS-NN; p<0.05). Only male pts. progressed to MM or other related diseases in the MGUS-N group (n=6, 100% vs. n=15, 47% in MGUS-NN; p<0.05).

12 pts. (32%) of the progressed 38 pts. had second tumors, thereof 11 pts. (34%) were in the MGUS-NN group and 1 pt. (17%) was in the MGUS-N group (p>0.05). 4 pts. had prostate cancer before diagnosis of MGUS, and all but two tumors developed before progression of MGUS.

Furthermore, in the total study population, pts. with a percentage of ≥5.5% plasma cells in the bone marrow had a significantly higher risk for progression than pts. with <5.5% plasma cells in the bone marrow (progression risk 38% vs. 7%, p<0.05).

## DISCUSSION

In this retrospective study, 223 MGUS pts. were evaluated in terms of neurological manifestations. 187 MGUS-NN pts. were compared to 36 MGUS-N pts. regarding clinical characteristics, disease progression and risk factors predicting progression to a malignant hematological disorder. The strengths of this study are the large number of included pts., all collected in the local database of our medical university, and the follow-up time of 10 years. Limitations existed in the partly incomplete documentation of pts.’ data intended for the analysis. Moreover, pts. treated at a University Hospital might create a selection bias in favor of MGUS-N pts.

The prevalence of several clinical and laboratory features (immunoglobulins, anemia, renal dysfunction, elevated calcium level, elevated β2-microglobulin, LDH level, incidence of second tumors, progression to different hematological diseases) differed between MGUS-NN and MGUS-N (Table 1). A significantly higher incidence of anemia was found in MGUS-NN. A potential explanation could be the significantly higher prevalence of IgG immunoglobulins in the MGUS-NN cohort, which can lead to monoclonal gammopathy of renal significance including chronic kidney disease with renal anemia [16, 17].

The annual risk of progression to MM in pts. with MGUS is about 1% [1]. The risk of progression to MM in pts. with light chain MGUS is 0.3% [2]. Our results confirmed these findings but differed between the two cohorts (progression rate 1% /year in the MGUS-N cohort vs. 0.5% /year in the MGUS-NN cohort), although the difference was not statistically significant. 38 of 223 pts. (17%) progressed to MM or other related hematological diseases (Figure 1). It was shown that pts. affected by MGUS-NN, progressed significantly more often to smoldering MM, MM and AL-amyloidosis compared to the MGUS-N group. By contrast, more progressions to Waldenstrom's macroglobulinemia were seen in the MGUS-N group. In particular, the immunoglobulin isotype, the M-protein concentration and the free light chains ratio are known to be risk factors related to progression [1, 4, 18]. Recently, Sigurdardottir and coworkers demonstrated that regular follow-up of MGUS pts. may be associated with the outcome in MM [19]. Thus, the guidelines recommend annual monitoring in high risk MGUS pts. and follow-up in low risk MGUS pts. presenting with clinical symptoms [4]. As optional investigation, in IgM-MGUS with higher risk for progression into Waldenstrom's macroglobulinemia than non-IgM MGUS, an evaluation of lymphadenopathy and/or spleen size by sonography could be done.

In our cohort, 36 pts. (16%) with MGUS had an associated neuropathy. This finding is in line with the study of Nobile-Orazio et al., who described 17% MGUS-N in their population (thereof 6% in IgG-MGUS, 14% in IgA-MGUS, and 31% in IgM-MGUS pts.) [5]. Axonal neuropathy was the most frequent entity among our pts. (55% of MGUS-N) and most of these cases probably had a coincidental association with MGUS not requiring specific treatment except agents for neuropathic pain [9, 20]. Some pts. with IgG and IgA paraproteinaemic axonal neuropathy, however, may benefit from immunotherapy [21], as was seen in two pts. from our cohort; therefore these pts. should not be a priori excluded from these treatment options.

IgM-MGUS has the highest prevalence in monoclonal gammopathy associated with neuropathy, that usually manifests with distal symmetric sensorimotor and atactic features [6, 22, 23]. These findings (IgM-MGUS-N in our total IgM cohort of 42 pts. was 31%) and the results of Kristinsson et al., that MGUS of IgM type is associated with a higher progression risk to Waldenstrom's macroglobulinemia [24], are confirmed by our study. In our cohort, all 6 pts. who progressed to Waldenstrom's macroglobulinemia had previously IgM-MGUS-NN or IgM-MGUS-N, respectively (see Table 3). The MGUS-N group showed significantly more progressions to Waldenstrom's macroglobulinemia compared to the MGUS-NN group (4 of 6 progressed pts. in MGUS-N; 67% vs. 2 of 32 progressed pts. in MGUS-NN; 6%; p<0.05). The disease course of neuropathy due to IgM-MGUS with or without antibodies against MAG is highly variable [25] and studies assessing the efficacy of intravenous immunoglobulins [26, 27] and Rituximab [28, 29] showed inconsistent results. In CIDP associated with monoclonal gammopathy, steroids, intravenous immunoglobulins and plasma exchanges represent the main therapeutic options as in idiopathic CIDP and 66-80% of pts. respond to one of these treatments [11, 30, 31]. On long-term follow-up, however, progression of functional deficits is greater in MGUS-CIDP pts. compared to idiopathic CIDP [32].

In our total study population, a threshold of ≥5.5% plasma cells in the bone marrow was found to be a strong predictor for disease progression. Although this was described previously [33, 34], nevertheless it is an important finding and should be evaluated in the future as a risk factor for disease progression. Hitherto, special molecular alterations and drivers for disease progression in MGUS and MM are still unknown, but it has been shown that changes in the bone marrow microenvironment develop early and consistently [35]. It might be suggested that clonal proliferation of plasma cells increases genetic instability with possible driver mutations in different subclones [36, 37]. The higher the clonal expansion, the higher the number of potential triggers. Therefore, in our opinion, for MGUS pts. with a percentage of ≥5.5% plasma cells in the bone marrow, a close-meshed monitoring to detect progression to MM or other related hematological disorders is recommended. However, a routine bone marrow examination in newly diagnosed MGUS pts. is not currently indicated.

In summary, in this study we analyzed the prevalence of neurological manifestations in MGUS pts. and demonstrated differences in clinical features and risk factors for disease progression in a large cohort of MGUS-NN and MGUS-N pts. In our study population, a considerable part of MGUS pts. (16%) had a neuropathy. Peripheral neuropathy associated with monoclonal gammopathy is a complex problem in clinical practice. As MGUS pts. frequently are not referred to a specialized center, that may result in an underestimation of neurological symptoms and their complications or in a non-detection of a correlation with MGUS-N disease. Therefore, it is important that MGUS pts. are monitored carefully and referred to a specialized center if neurological symptoms occur, particularly those with progressive sensorimotor deficits and ataxia. Furthermore, due to the risk of progression to MM or other related diseases (17% of the pts. in both groups), the important goal for MGUS pts. must be the detection of an early progression into MM and the prevention of complications. MGUS pts. should be considered for risk-and-response stratified therapy monitoring even in terms of neurological manifestations, and an early supportive treatment should be conducted to improve their quality of life, as well as immunomodulatory and /or specific treatments in pts. with progressive neuropathies.

## PATIENTS AND METHODS

Between January 2005 and March 2015, a total of 223 adult pts. aged between 26 and 97 years with MGUS according to the International Myeloma Working Group (IMWG) criteria [3], were identified by an exploration in our local database. Observation period in some pts. lasted over 10 years since the initial diagnosis was made before 2005. Sampling and evaluation of pts.’ data was approved by the local institutional ethics committee (vote number: AN2015-0193 352/4.13) and was conducted in accordance with the principles of the Declaration of Helsinki. The study included pts.’ data about the clinical presentation, physical examination and laboratory tests. Two cohorts, MGUS-NN and MGUS-N pts., were compared and were analyzed for disease progression. “Progression” is used in the whole paper as term for every change into other diseases. MGUS-N pts. were diagnosed by a consultant neurologist or referred to the neuromuscular unit of the Department of Neurology and classification of neuropathy was based on standard electrophysiological studies.

Statistical evaluation was performed using SPSS statistical software (version 20.0; SPSS Inc., Chicago, IL, USA). All tests for statistical significance were two-sided. Chi-squared test, unpaired t-test and survival analysis (Kaplan Meier curves, log-rank test) were used to identify differences between two groups. A p-value of <0.05 was considered as statistically significant.
